# AI literacy and creative self-beliefs among Chinese college students: examining the underlying mechanisms and boundary conditions

**DOI:** 10.3389/fpsyg.2026.1719970

**Published:** 2026-02-04

**Authors:** Cong Peng, Zhenlin Zhang, Zhenyun Zhang, Jie Zhang, Ziyi Zhang, Yuan Liu, Rui Jia, Xiaoyang Xu

**Affiliations:** 1 School of Education and Psychology, Hubei Engineering University, Xiaogan, China; 2 School of Computer and Information Science, Hubei Engineering University, Xiaogan, China

**Keywords:** AI literacy, college students, creative self-beliefs, frequency of AI usage, problem-solving ability

## Abstract

**Introduction:**

With the rapid advancement of artificial intelligence (AI), enhancing AI literacy has become a critical objective in higher education. How to leverage AI to foster students’ creativity thus emerged as a research focus. Creative self-beliefs (creative self-efficacy and creative personal identity) serve as a key predictor of individual creativity and innovative behavior. However, little is known about the direct and indirect associations of AI literacy on creative self-beliefs among college students.

**Methods:**

Based on self-efficacy theory, social cognitive theory, and cognitive load theory, this study investigated the relationship between college students’ AI literacy and their creative self-beliefs, constructing a moderated mediation model. Standardized scales were employed to survey 644 Chinese college students.

**Results:**

(1) College students’ AI literacy significantly and positively predicted their creative self-beliefs, and problem-solving ability partially mediated the relationship between them. The mediating effect of problem-solving ability accounted for 64.2% of the total effect. (2) Frequency of AI usage moderated both the direct path from AI literacy to creative self-beliefs and the path from problem-solving ability to creative self-beliefs.

**Discussion:**

These findings provide empirical evidence elucidating how AI literacy influences Chinese college students’ creative self-beliefs, highlighting the necessity of aligning AI utilization with core competencies to maximize its value. The study concludes with recommendations for promoting critical engagement with AI tools to fully leverage the technology’s potential for enhancing college students’ confidence in innovation.

## Introduction

With continuous advancements in artificial intelligence (AI) tools, the rapid development of generative AI is reshaping higher education. AI literacy refers to an individual’s ability to understand, apply, and critically evaluate AI technologies (Ma and Chen, 2024). Enhancing AI literacy has become a critical objective in higher education. Research indicates that AI literacy significantly affects university students’ English learning outcomes (Zhang Q. et al., 2025), academic stress (Ibrahim et al., 2024), employee job performance (Liu et al., 2025), innovative and creative thinking (Agaoglu et al., 2025), and job-seeking anxiety (Li et al., 2025).

As AI evolves, leveraging it to enhance students’ creativity has become a critical research focus. Over the last 20 years, psychological studies have increasingly focused on certain personal factors that support creativity, known as creative self-beliefs. Among several creative self-beliefs, two in particular have dominated the research of the past 10 years: creative self-efficacy and creative personal identity (Karwowski et al., 2018). The creative self-efficacy (CSE) refers to an individual’s beliefs in their ability to generate creative ideas and achieve creative outcomes (Puente-Díaz, 2016). The creative personal identity (CPI) is one of creative self-image beliefs, referring to the extent to which creativity is treated as an important part of an individual’s sense of self (Lebuda et al., 2020). As a key predictor of creative behavior (e.g., engage in creative tasks or activities, employ creativity in areas of life), high creative self-efficacy (CSE, as part of creative self-beliefs) fosters confidence in creative activities, sustains creative efforts, and drives successful innovation (Li et al., 2022; Tierney and Farmer, 2011). Studies showed that CSE was positively correlated with creativity among university students (Yang et al., 2020). Moreover, the high value inscribed to creativity (CPI, as part of creative self-beliefs) is one of the main engines of beginning a creative activity (Kasof et al., 2007). The extent to which one values creativity is a key determining factor in the choice to pursue creative activities (Sternberg, 2002). Individuals for whom creativity is part of their self-definition will seek out opportunities to be creative at work in order to maintain positive self-regard and affirm a key part of their self-concept (Steele, 1988). Thus, both parts of CSB serves as crucial determinants of creativity.

People will only act creatively if they are both confident in their abilities and value the importance of creativity (Karwowski and Beghetto, 2019). CSE and CPI are closely linked yet distinct constructs (Jaussi et al., 2007). While creative self-efficacy is understood to mediate the link between creative potential and creative action, creative personal identity functions more as a moderator of this connection (Karwowski et al., 2018). The two also exhibit a reciprocal influence on one another (Karwowski, 2016), and they form a higher-order construct known as the creative self (Karwowski, 2012). Therefore, drawing on the theoretical framework of the Creative Self Scale developed by Karwowski et al. (2018) and recent research on creative self-beliefs (e.g., de Oliveira Jorge and de Souza Fleith, 2023; Pavlić et al., 2023), this study will focus on examining two related dimensions of creative self-beliefs: creative self-efficacy and creative personal identity.

Factors influencing creative self-beliefs (creative personal identity and creative self-efficacy) among college students have been extensively investigated in previous research. Existing studies primarily focus on exploring the factors influencing creative self-beliefs from three aspects: individual internal factors, external environmental factors, and generative AI-based technologies. Individual internal factors include curiosity (Puente-Díaz and Cavazos-Arroyo, 2017), emotional intelligence (Chen and Cheng, 2023), thinking styles (Li and Liu, 2014), cognitive load management (Redifer et al., 2021), and awareness of future challenges (Alt et al., 2023). External environmental factors encompass student collaboration (Nja et al., 2023), teachers’ knowledge innovation (Chang, 2018), evidence-based practice climate in education (El-Sayed A. A. I. et al., 2025), as well as parental educational expectations and parent–child interactions (Abaszadeh et al., 2024). Furthermore, generative AI-based technologies have been shown to affect university students’ self-efficacy, which in turn influences their academic performance and creativity (Shahzad et al., 2024).

To date, although recent empirical research has developed a model illustrating the impact of generative AI-based technologies on Chinese university students’ academic performance, with general self-efficacy playing a significant mediating role (Shahzad et al., 2024), studies have predominantly focused on the effect of AI-related technical skills while neglecting the role of AI literacy in higher-order cognitive abilities. Research on the influence of AI literacy on university students’ creative self-beliefs remains relatively scarce. The direct and indirect relationships between AI literacy and creative self-beliefs among college students remain underexplored. Although recent studies have demonstrated that AI literacy can influence employees’ work performance through its effect on creative self-beliefs (Liu et al., 2025), and has shown significant predictive power for nursing students’ career and talent self-efficacy (El-Sayed B. K. M. et al., 2025), the mechanisms through which AI literacy affects college student’ creative self-beliefs require further investigation. Additionally, it remains unclear whether frequency of AI usage leads to technological dependency or serves to empower and reshape student’ innovative capacities.

This study seeks to address these research gaps by developing an integrated theoretical framework that combines key constructs from self-efficacy theory (Kleppang et al., 2023), social cognitive theory (Bandura, 2001), and attribution theory (Thatcher et al., 2008), while incorporating unique factors related to creative self-beliefs in the context of AI utilization. By doing so, this study advances both theoretical understanding and practical applications through three key contributions. First, it pioneers a novel conceptualization of AI literacy as a catalyst for developing creative self-beliefs, establishing an important link between technical proficiency and psychological empowerment in digital learning environments. Second, it unveils the complex mechanisms underlying the relationships among AI literacy, problem-solving ability, frequency of AI usage, and creative self-beliefs through an empirically validated moderated mediation model, addressing a significant research gap in digital competence studies. Third, the findings provide guidelines for developing targeted educational interventions that simultaneously enhance students’ technical AI skills and confidence in creative solutions. These insights not only enrich social cognitive theories in technology-enhanced learning contexts but also equip educators with a robust framework to foster AI-driven innovation in higher education, paving the way for future research at the intersection of AI literacy development and creative competency cultivation.

### AI literacy and creative self-beliefs

Firstly, based on self-efficacy theory, self-efficacy is significantly influenced by one’s past successful experiences (Kleppang et al., 2023). Equipped with AI literacy, college students can leverage it to streamline creativity, from inspiration to rapid prototyping. These capabilities enable them to accumulate positive experiences and strengthen their confidence in their innovative abilities. By reducing barriers to creativity, AI literacy empowers individuals to express ideas more effectively. As students achieve tangible creative outcomes through AI-assisted methods, their sense of creative self-beliefs becomes reinforced. Research found that AI applications boosted creativity through the introduction of new ideas and problem-solving techniques (Lin and Chen, 2024), although it faces significant challenges that need to be confronted, including creativity constraints, emotional disengagement, and performance anxiety. Therefore, the extent and nature of AI applications’ role in stimulating creativity still require comprehensive consideration of other factors.

Secondly, the relationship between the two can be explained not only by self-efficacy theory but also through the complementary lens of social cognitive theory. According to social cognitive theory (Bandura, 2001), an individual’s self-efficacy is shaped by the interaction among personal abilities, the environment (e.g., AI tools), and behaviors (e.g., methods of AI usage). Enhanced AI literacy increases a sense of control. College students who master AI tools are more confident in managing technology-assisted creative tasks, which reduces anxiety about unfamiliar technologies and boosts confidence in creative solutions. The study found that students with high AI literacy are better able to utilize AI tools for creative writing and visual storytelling, as AI can help overcome creative blocks, stimulate new ideas, and enhance creative autonomy (Tsao and Nogues, 2024).

Based on this, the study proposes:

*H1:* AI literacy significantly positively predicts college students’ creative self-beliefs.

### The mediating role of problem-solving ability between AI literacy and CSB

First, the enhancement of AI literacy facilitates the improvement of college students’ problem-solving ability. Problem-solving ability refers to an individual’s comprehensive capacity to identify issues, analyze information, formulate strategies, and effectively implement solutions in complex situations (Thabet et al., 2017). Research showed individuals with high AI literacy can transform real-world problems into computable models, thereby enhancing structured analytical skills and problem-solving competence of students (Kong et al., 2024). AI significantly aids college students in addressing real-world problems. For instance, AI tools can rapidly process complex data, reduce cognitive load, and enable individuals to focus more on higher-order strategic planning (Mehta et al., 2025). AI assistance can provide diverse perspectives, breaking through traditional cognitive limitations. Empowerment theory posits that AI literacy enhances problem-solving ability by augmenting individuals’ cognitive resources, such as information processing efficiency and knowledge integration capabilities (Wang et al., 2022). In terms of information acquisition and processing, college students with high AI literacy can effectively utilize AI tools to retrieve, filter, and synthesize information, thereby reducing cognitive load in writing tasks and allowing them to concentrate on problem analysis (Pellas, 2023). Regarding knowledge transfer and application, AI-assisted learning helps college students identify knowledge gaps, reduce cognitive anxiety, and bolster confidence in problem-solving. Research found that personalized learning pathways further enhance college students’ transfer of problem-solving skills across contexts (Lin and Chen, 2024). Therefore, the rational and effective application of AI empowers university students to improve their problem-solving capabilities in scholarly and everyday life.

Moreover, self-efficacy theory suggests that self-efficacy reflects an individual’s belief in their ability to complete specific tasks or achieve particular goals (Klassen and Klassen, 2018). When college students successfully solve problems, their self-beliefs is reinforced (Wang et al., 2024). Beliefs about one’s capabilities (e.g., creative self-efficacy) are shaped by prior successful experiences. Problem-solving ability embodies these successful experiences, strengthening students’ confidence in their creative abilities. Study found that using AI to solve problems efficiently can reduce anxiety and encourage Chinese college students to take more innovative risks (Hwang and Wu, 2025). Successful problem-solving leads to increased self-efficacy regarding innovation (Wu and Zhang, 2025).

Besides, college students with higher AI literacy can more effectively utilize AI tools to support learning and problem-solving, thereby enhancing their confidence and self-efficacy (Bewersdorff et al., 2025; Shahzad et al., 2024). Students with this confidence experience less technology-related stress, allowing them to readily approach complex tasks and develop successful outcomes that confirm their capacity to find solutions (Kong et al., 2024; Bécue et al., 2024). Therefore, successful problem-solving experiences with AI tools can strengthen college students’ self-efficacy, creating a positive feedback loop.

Based on this reasoning, we propose:

*H2:* Problem-solving ability mediates the relationship between AI literacy and college students’ creative self-beliefs.

### The moderating role of frequency of AI usage

While AI literacy may enhance university students’ creative self-beliefs (CSB) through cognitive empowerment and successful experiences, overdependence on AI may undermine students’ confidence in their own abilities and stifle creativity through standardized solutions. Existing research has largely overlooked the crucial role of frequency of AI usage in the pathway between AI literacy and CSB. Recent studies have found that AI is a double-edged sword, and AI usage may have varying effects on users’ psychology and behavior (Lin and Chen, 2024; Liu and Li, 2025). AI literacy refers to an individual’s capability to understand, apply, and critically evaluate AI technologies. Whether this capability can translate into a form of self-belief may be moderated by AI usage behaviors, such as frequency of usage. Frequency of AI usage may serve as a moderator in the direct path (AI literacy → CSB). As AI usage frequency increases, the positive effect of AI literacy on college students’ CSB may diminish.

According to self-efficacy theory, CSB (especially the creative self-efficacy) is influenced by autonomy and competence perceptions (Liu et al., 2021). Research indicates that students’ engagement and self-efficacy in e-learning are positively associated with perceived autonomy (Huang and Liaw, 2007). Over-reliance on AI may reduce autonomy, thereby lowering CSB. Moderate usage enhances CSB by maintaining autonomous thinking and reinforcing competence perceptions. Conversely, excessive usage allows AI to dominate problem-solving, undermining college students’ autonomy and consequently reducing CSB. Cognitive load theory also suggests that students’ cognitive engagement is influenced by environmental perceptions (Mehta et al., 2025). Thus, AI’s effectiveness depends on users’ cognitive capacity. Heightened AI usage may increase cognitive load for students with lower AI literacy, impeding their ability to effectively leverage AI for learning optimization. High-AI-literacy students might reduce deep thinking through over-reliance. Research shows that while AI-assisted language learning enhances emotional engagement, excessive dependence reduces cognitive investment (Alrashidi and Alshammari, 2024). Positive academic emotions correlate with engagement, but AI overuse may impair learning outcomes.

Based on these insights, we propose:

*H3:* Frequency of AI usage moderates the direct path between AI literacy and creative self-beliefs.

Frequency of AI usage may also moderate the relationship between problem-solving ability and CSB. Students with strong problem-solving skills can utilize AI effectively without over-reliance, thereby maintaining or enhancing CSB. Evidence suggests that individuals adept at metacognitive regulation, which involves actively managing their learning and reasoning, are more effective at utilizing AI assistance to expand cognitive capacity, thus fostering creativity (Sun et al., 2025). Conversely, those with weaker skills may become dependent, reducing autonomous thinking and undermining CSB (He, 2025).

Cognitive offloading theory posits that over-reliance on external tools may degrade internal cognitive abilities (Risko and Gilbert, 2016). Students with solid problem-solving skills are so secure in their abilities that they can use AI as a supplement without it shaking their creative self-beliefs. However, for low-ability students, frequent AI use may weaken their independent thinking and problem-solving skills, lowering their confidence in creative solutions.

Therefore, we propose:

*H4:* Frequency of AI usage moderates the latter path between problem-solving ability and creative self-beliefs.

In summary, this study integrates multiple classic theories to construct a comprehensive analytical perspective of individual experience and cognitive interaction, aiming to systematically analyze the internal mechanisms and boundary conditions through which AI literacy influences creative self-beliefs. Regarding the relationship between AI literacy and creative self-beliefs, Self-efficacy Theory emphasizes the sources of individuals’ mastery experiences, while Social Cognitive Theory highlights the triadic interaction between environment, behavior, and the individual. These two theories help us elucidate why AI literacy serves as both a “source of successful experiences” and an “environmental enabling factor” for creative self-beliefs. Concerning the mediating role of problem-solving ability between AI literacy and creative self- beliefs, this study draws on Empowerment theory and Cognitive Load Theory to analyze it from two complementary perspectives. Through the joint mechanism of cognitive empowerment and the reduction of anxiety, AI literacy can enhance individuals’ creative self-beliefs by improving their problem-solving ability. As for the moderating role of AI usage frequency, the analysis is conducted primarily from two dimensions. At the belief level, Self-efficacy Theory posits that perceptions of autonomy and competence are the most powerful sources of individual’s creative self-beliefs. This sheds light on the process whereby frequent use of AI for solving tasks may affect an individual’s autonomy, which in turn shapes their creative self-beliefs. At the cognitive level, Cognitive Offloading Theory suggests that offloading cognitive tasks to AI tools can free up an individual’s limited cognitive resources, allowing them to focus their most valuable core cognitive resources on the most creative aspects of problem-solving, thereby enhancing creative self-beliefs. For individuals with strong problem-solving abilities, frequency of AI usage further amplifies the benefits derived from such cognitive offloading.

## Current research

**FIGURE 1:**
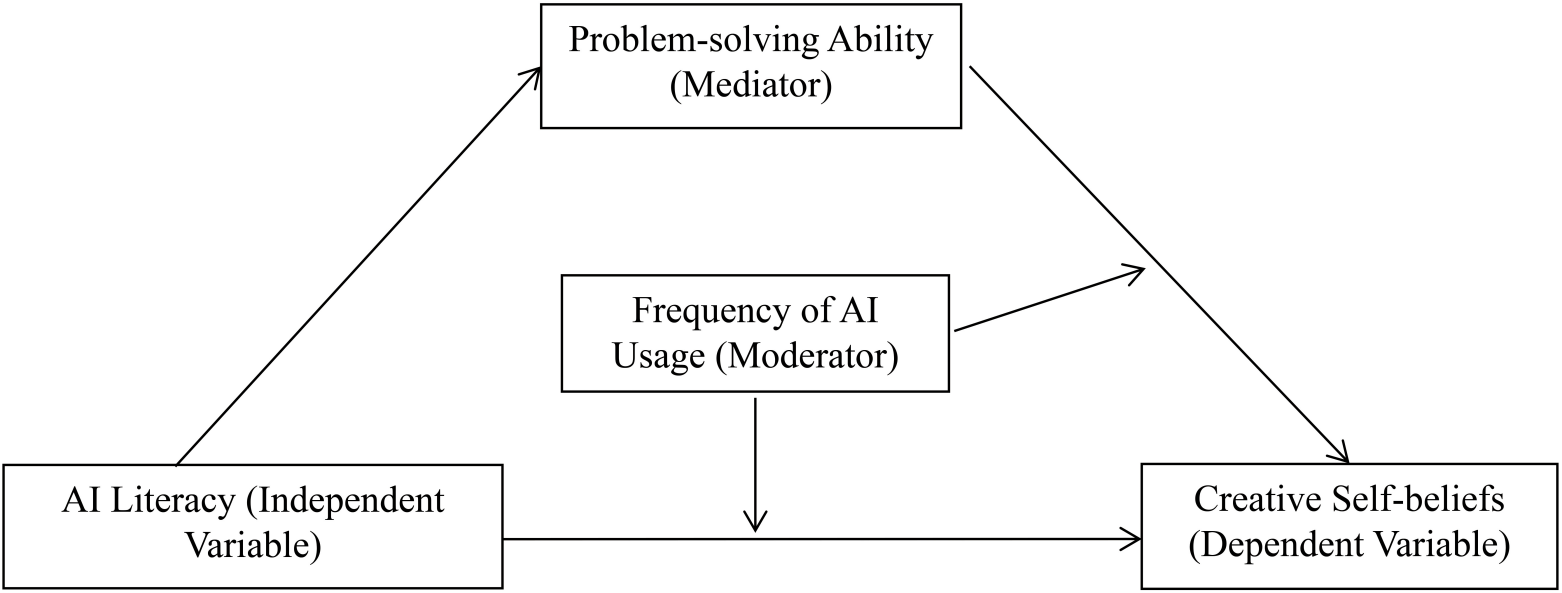
The proposed moderated mediation model.

Prior research has largely overlooked both the impact of AI literacy on college students’ creative self-beliefs and the mechanisms underlying this relationship. To address this gap, the current study proposes a moderated mediation model (Figure 1). Grounded in Self-Efficacy Theory, Social Cognitive Theory, and Cognitive Load Theory, we conducted an empirical study to examine the following hypotheses: (1) Hypothesis 1: AI literacy significantly positively predicts college students’ creative self-beliefs; (2) Hypothesis 2: Problem-solving ability mediates the relationship between AI literacy and college students’ creative self-beliefs; and (3) Hypothesis 3–4: Frequency of AI usage moderates both the direct path between AI literacy and creative self-beliefs and the latter path between problem-solving ability and creative self-beliefs.

## Materials and methods

### Participants

A convenience sampling approach was employed across four universities in Hubei and Henan Provinces, China, yielding 684 submitted online questionnaires. The universities in our sample are primarily local ordinary undergraduate institutions, rather than China’s prestigious 985 or 211 Project universities. There are significant distinctions between Chinese 985/211 Project universities and local ordinary undergraduate institutions. The former, primarily serving regional development needs, are more constrained in resource allocation, research capacity, and admission thresholds, with a stronger focus on cultivating applied talents. The survey was administered electronically via questionnaires edited with WenJuanXing software in classroom settings. Invalid questionnaires were excluded due to incompleteness, uniform response patterns, or abnormally short completion times. After quality checks, 644 responses were retained as valid, representing a 94.2% response rate. The final sample (*N* = 644) comprised students aged 16–28 years (*M* = 19.62, *SD* = 1.24), with 270 males (41.9%) and 374 females (58.1%). Academic year distribution included 280 freshmen (43.5%), 160 sophomores (24.8%), and 204 juniors (31.7%). Additionally, 338 students are from humanities and social sciences disciplines (52.5%), while 306 are from science and engineering disciplines (47.5%). 64 participants (9.9%) were vocational college students, while 580 (90.1%) were undergraduates.

### Measures

#### AI literacy scale

The AI Literacy Scale for Chinese College Students (AILS-CCS) developed by Ma and Chen (2024) was adopted, comprising 15 items across four dimensions: Awareness (4 items, e.g., I understand the definition of artificial intelligence.), Usage (3 items, e.g., I can use artificial intelligence applications or products to assist my learning.), Evaluation (4 items, e.g., I remain skeptical or cautious about content generated by artificial intelligence.), and Ethics (4 items. e.g., I am always alert to the misuse of artificial intelligence technology.). Responses were recorded on a 5-point Likert scale, with higher scores indicating greater AI literacy. The scale demonstrated excellent internal consistency in this study (Cronbach’s α = 0.919).

#### Creative self-scale

Creative self-beliefs were measured using the scale developed by Karwowski et al. (2018), which consists of 11 items. Six items measure creative self-efficacy (e.g., I am sure I can deal with problems requiring creative thinking). Five items are dedicated to assessing creative personal identity (e.g., My creativity is important to who I am). Responses were collected using a 5-point scale (1 = definitely not to 5 = definitely yes), with higher scores reflecting greater creative self-beliefs. The scale showed outstanding reliability (Cronbach’s α = 0.950).

#### Problem-solving inventory

The Chinese version of the Social Problem-Solving Inventory developed by Siu and Shek (2005) was employed, originally containing 24 items across five dimensions. For this study, we selected two subscales: Rational Problem Solving and Positive Problem Orientation (10 items total). Sample items include “I use a systematic method for comparing alternatives.” (Rational Problem Solving) and “I do not give up trying to solve problems when first attempt fails” (Positive Problem Orientation). The adapted scale exhibited excellent reliability (Cronbach’s α = 0.932).

#### Frequency of AI usage

Frequency of AI usage was measured using a single-item indicator from the AILS-CCS (Ma and Chen, 2024), assessing weekly AI usage with four response options: 0 times, 1–2 times, 3–4 times, and 5++ times per week. Higher scores indicated more frequent AI usage.

#### Procedure

Data collection through online software was conducted in university classroom settings between March 13 and May 14, 2025. Prior to participation, all students provided written informed consent in accordance with the Declaration of Helsinki, and the study protocol was approved by the Ethical Committee of the School of Education and Psychology at Hubei Engineering University. Participants were explicitly informed of the voluntary nature of their involvement and their right to withdraw at any time without penalty. Six trained college instructors facilitated the data collection process by administering standardized instructions, distributing online questionnaire links, addressing participant queries, and ensuring timely completion. The survey required approximately 10–15 min to complete.

#### Data analysis

The statistical analyses were conducted with SPSS 23.0, beginning with descriptive statistics and bivariate correlations. Intervariable relationships were examined using Pearson correlation coefficient, revealing statistically significant correlations between independent variable, mediating variable, and the dependent variable—a prerequisite for mediation analysis. Building on these findings, the study employs PROCESS macro’s regression-based approach to test a moderated mediation model (Hayes, 2012). The analysis will investigate the mediating roles of problem-solving ability between AI literacy and students’ creative self-beliefs, and the moderating effects of frequency of AI usage across different pathways in the proposed model. The analytical approach involved two distinct models: Model 4 tested the mediating role of problem-solving competence in linking AI literacy to creative self-beliefs in the student sample, while Model 15 assessed how this mediation process varied across different levels of frequency of AI usage. A bootstrap analysis with 5,000 replications was conducted to evaluate the significance of all effects and obtain robust parameter estimates. This method also enabled the estimation of the 95% confidence interval for the effect (Zhang et al., 2024).

#### Common methodological biases

Given that the data in this study were collected solely through self-report measures from college students, potential common method bias was rigorously evaluated. To assess this issue, Harman’s single-factor test was conducted. The results of the unrotated principal component analysis revealed five distinct factors with eigenvalues > 1. Importantly, the first factor accounted for only 15.81% of the total variance, falling well below the critical threshold of 40%. These findings indicate that common method bias does not pose a substantial concern in the present study (Podsakoff et al., 2003).

## Results

### Preliminary analysis

Table 1 presents the descriptive statistics (means and standard deviations) and bivariate correlations among all study variables. The correlation analysis revealed three significant findings: (1) both AI literacy and its four dimensions demonstrated positive associations with creative self-beliefs (*P* < 0.01), and problem-solving ability (*P* < 0.01); (2) creative self-beliefs showed a significant positive relationship with problem-solving ability (*P* < 0.01); and (3) AI literacy and problem-solving ability were positively correlated with frequency of AI usage (*P* < 0.05). While these correlational patterns provide preliminary evidence for the interrelationships among variables, more rigorous testing is warranted to establish the precise mediation pathways.

**TABLE 1:** The correlation of the main study variables (*N* = 644).

Variables	*M* ± *SD*	1	2	3	4	5	6	7	8	9	10
1 AI Awareness	3.30 ± 0.76	1									
2 AI usage	3.81 ± 0.66	0.47**	1								
3 AI evaluation	3.60 ± 0.63	0.58**	0.71**	1							
4 AI ethics	3.73 ± 0.65	0.50**	0.61**	0.72**	1						
5 AI literacy	3.60 ± 0.57	0.80**	0.80**	0.89**	0.85**	1					
6 Problem-solving ability	3.79 ± 0.58	0.40**	0.52**	0.59**	0.61**	0.63**	1				
7 Creative self-beliefs	3.55 ± 0.67	0.44**	0.45**	0.54**	0.49**	0.58**	0.70**	1			
8 Frequency of AI usage	3.30 ± 0.89	0.03	0.15**	0.10*	0.05	0.09*	0.09*	0.03	1		
9 Age	19.62 ± 1.24	-0.06	0.01	0.05	0.02	0	0.06	0.01	-0.05	1	
10 Gender	0.58 ± 0.49	-0.17**	0.03	-0.01	-0.02	-0.06	0.06	–0.06	0.13**	0.03	1

*P< 0.05,

**P < 0.01.

### Testing for mediation effect of problem-solving ability

The correlation analysis presented in Table 1 demonstrates statistically significant relationships between three key variable pairs: AI literacy and creative self-beliefs, AI literacy and problem-solving ability, and problem-solving ability and creative self-beliefs. These significant associations between independent and mediating variables satisfy the prerequisite conditions for mediation analysis. To rigorously examine these relationships, we employed the PROCESS macro program’s Model 4 (simple mediation model) using the bias-corrected bootstrap method. This analytical approach generated 5,000 bootstrap samples to calculate robust estimates of the mediation effects, with 95% confidence intervals providing the statistical precision for our findings.

First, we conducted a mediation analysis controlling for gender, age, and grade, with AI literacy as the independent variable, problem-solving ability as the mediator, and creative self-beliefs as the dependent variable. The regression results presented in Table 2 demonstrate that the positive predictive effect of AI literacy on creative self-beliefs was significant (*B* = 0.57, *t* = 17.71, *P* < 0.001). When the mediating variable of problem-solving ability was added, the direct predictive effect of AI literacy on creative self-beliefs was still significant *(B* = 0.21, *t* = 5.85, *P* < 0.001). The positive predictive effect of AI literacy on problem-solving ability was significant (*B* = 0.63, *t* = 20.77, *P*< 0.001), and the positive predictive effect of problem-solving ability on creative self-beliefs was also significant (*B* = 0.58, *t* = 16.53, *P* < 0.001).

**TABLE 2:** Testing the mediation effect of AI literacy on creative self-beliefs.

Regression equation (*N* = 644)		Fitting indicators			Coefficient significance	
Outcomes	Predictors	*R*	*R* ^2^	*F*	β	*t*
Creative self-beliefs		0.58	0.33	79.45***		
	Gender				-0.06	-0.92
	Age				-0.01	-0.23
	Grade				0.02	0.41
	AI literacy				0.57	17.71***
Problem-solving ability		0.64	0.41	110.12***
	Gender				0.20	3.10**
	Age				0.05	1.47
	Grade				-0.01	-0.20
	AI literacy				0.63	20.77***
Creative self-beliefs		0.73	0.53	145.31***		
	Gender				-0.18	-3.10**
	Age				-0.04	-1.23
	Grade				0.03	0.62
	AI literacy				0.21	5.85***
	problem-solving ability				0.58	16.53***

**P< 0.01,

***P < 0.001.

The mediation analysis results presented in Table 3 further demonstrate the relationship between AI literacy and creative self-beliefs. The bootstrap analysis with 95% confidence intervals confirmed the significance of problem-solving ability’s mediating role, as the interval excluded zero. Quantitative decomposition revealed that the direct effect (0.206, 35.8%) and mediating effect (0.369, 64.2%) collectively accounted for the total effect (0.575). These findings provide empirical evidence for problem-solving ability’s partial mediation in the relationship between AI literacy and creative self-beliefs.

**TABLE 3:** The estimates of total, direct and indirect effects of the model.

Effect		Boot SE	Boot LLCI	Boot ULCI	Relative effect value
Total effect	0.575	0.032	0.511	0.638	
Direct effect	0.206	0.035	0.137	0.274	35.8%
Indirect effect of PSA	0.369	0.039	0.293	0.447	64.2%

PSA, Problem-solving ability.

### Testing for moderated mediation effect

The moderated mediation analysis utilized Hayes’ PROCESS Model 15 in SPSS, specifically designed to examine moderation effects on both the direct path (AI literacy → creative self-beliefs) and the second stage of mediation (problem-solving ability → creative self-beliefs), aligning with the study’s theoretical framework. The results (see Table 4) show that frequency of AI usage has a moderating effect on the direct prediction of creative self-beliefs by AI literacy (*B* = –0.07, *t* = –2.044, *P*<0.05), and it can also moderate the prediction effect of problem-solving ability on creative self-beliefs (*B* = 0.07, *t* = 2.18, *P*< 0.05).

**TABLE 4:** Testing the moderated mediation effect of AI literacy on creative self- beliefs.

Regression equation (*N* = 644)		Fitting indicators			Coefficient significance	
Outcomes	Predictors	*R*	*R* ^2^	*F*	β	*t*
Problem-solving ability		0.64	0.41	110.12***		
	Gender				0.20	3.10**
	Age				0.05	1.47
	Grade				-0.01	-0.20
	AI literacy				0.63	20.77***
Creative Self-beliefs		0.73	0.54	92.15***		
	Gender				-0.17	-2.99**
	Age				-0.04	-1.16
	Grade				0.03	0.62
	AI literacy				0.20	0.04***
	Problem-solving ability				0.59	16.74***
	Frequency of AI usage				-0.03	-1.17
	AI literacy × Frequency of AI usage				-0.07	-2.04*
	Problem-solving ability × Frequency of AI usage				0.07	2.18*

*P< 0.05,

**P< 0.01,

***P < 0.001.

**FIGURE 2:**
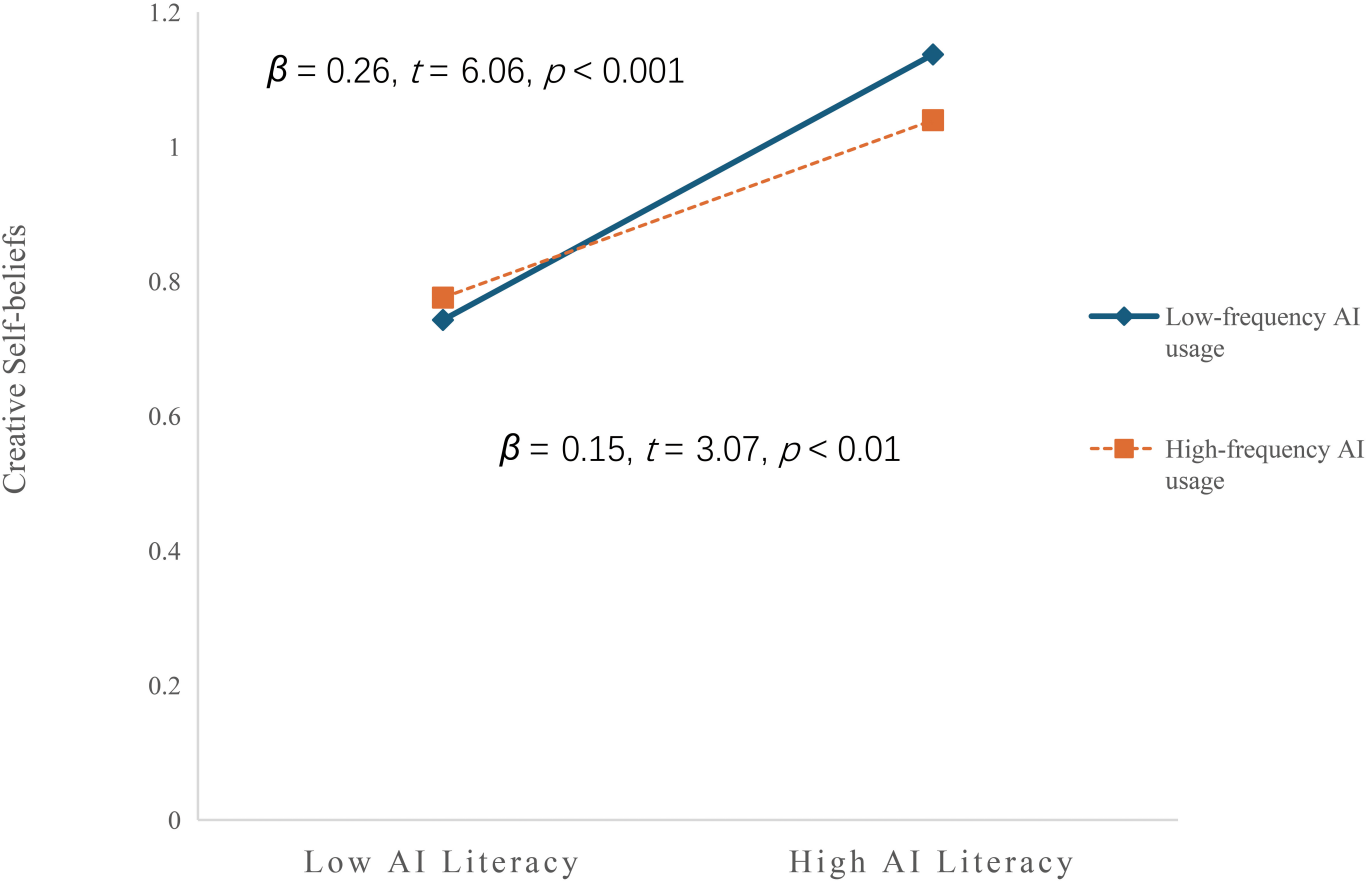
Moderating effect of frequency of AI usage between AI literacy and creative self-beliefs.

For enhanced interpretation of the moderated mediation effects, frequency of AI usage was categorized into high and low groups based on ± 1 standard deviation from the mean. Simple slope analysis was subsequently conducted to examine the conditional effects of AI literacy on creative self-beliefs across different levels of frequency of AI usage. The moderation analysis revealed a significant attenuation effect. Specifically, the positive association between AI literacy and creative self-beliefs weakened with increasing frequency of AI usage, as evidenced by the reduction in regression coefficients from 0.26 (*t* = 6.06, *P* < 0.001) at low frequency to 0.15 (*t* = 3.07, *P*< 0.01) at high frequency levels (Figure 2).

**FIGURE 3:**
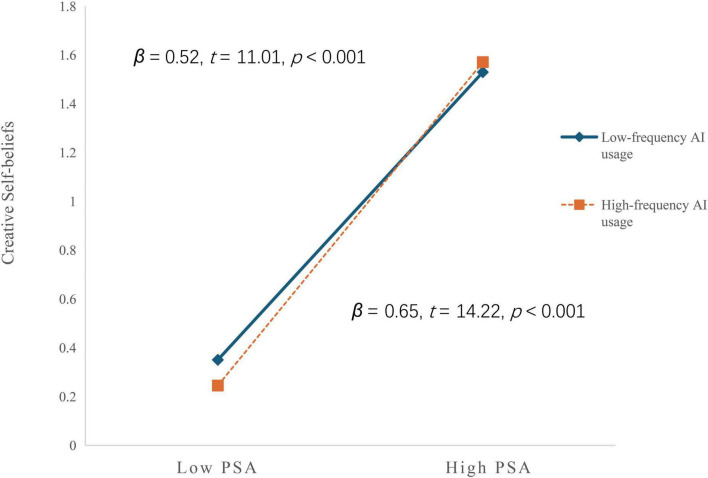
Moderating effect of frequency of AI usage between PSA and creative self- beliefs. PSA, problem-solving ability.

Parallel analysis of problem-solving ability’s predictive effects demonstrated an opposite pattern. The relationship between problem-solving ability and creative self-beliefs strengthened progressively with higher frequency of AI usage, with regression coefficients increasing from 0.52 (*t* = 11.01, *P*< 0.001) to 0.65 (*t* = 14.22, *P*< 0.001) across low to high frequency conditions (Figure 3).

## Discussion

This study constructed and tested a moderated mediation model. By integrating Self-Efficacy Theory, Empowerment theory, Cognitive Load Theory, Attribution Theory, and the Expertise Reversal Effect, we collectively depict a comprehensive picture of how AI literacy contributes to the formation of an individual’s creative self-beliefs, encompassing their relationship, underlying influence mechanisms, and boundary conditions. Specifically, Self-Efficacy Theory would explain why and how AI literacy serves as the initial driver of creative self- beliefs. Empowerment theory and Cognitive Load Theory elucidate the dual role of problem-solving ability in this process, involving both cognitive empowerment and the balance of cognitive load. Meanwhile, Self-Efficacy Theory, the Expertise Reversal Effect, and Attribution Theory clarify how the frequency of AI usage moderates the above relationships by altering individual autonomy and reinforcing attribution patterns. This study not only deepens our understanding of the psychological mechanisms of creativity in the AI era but also provides specific theoretical guidance for educational practices aimed at stimulating students’ innovative potential through the cultivation of AI literacy.

### The impact of AI literacy on college students’ creative self-beliefs

This study supports Hypothesis 1, indicating that AI literacy significantly and positively predicts college students’ creative self-beliefs (creative self-efficacy and creative personal identity), which aligns with recent research. AI literacy significantly and positively predicts university students’ creative personal identity (Li et al., 2026). Enhanced AI literacy enables students to better address challenges in learning and work, thereby strengthening their AI self-efficacy (Bewersdorff et al., 2025), which is consistent with conclusions from prior technology-enhanced learning studies (Waheed et al., 2020). Technology-enhanced learning emphasizes using technological tools to optimize the learning process and improve outcomes.

First, according to Self-efficacy Theory, individuals’ beliefs in their creative abilities stem from successful experiences and cognitive resources (Joët et al., 2011). Cultivating AI literacy equips students with greater competence in addressing challenges creatively, thereby accumulating more successful experiences (Tang et al., 2024). Cognitive resources encompass knowledge, skills, and strategies, and improved AI literacy helps students utilize these resources more effectively, bolstering their confidence in creative performance. Second, according to the technology acceptance model (TAM) and the empowerment effect, the effectiveness of technology usage is contingent upon users’ perceptions of the tool’s usefulness and ease of use (Lee J. W. Y. et al., 2025). Students with high AI literacy exhibit greater technology acceptance. The more familiar students are with AI, the better they can unlock its creative potential, thus enhancing their confidence in their own creative abilities. Research confirmed that college students’ technology acceptance is positively associated with both learning self-efficacy and the development of self-directed learning competencies (Pan, 2020). Recent study also demonstrated that participants significantly improved their efficiency in completing creative tasks after engaging in deep collaborative and innovative dialogues with AI (Lee S. H. et al., 2025).

As an emerging technology, AI presents new opportunities for education (Shahzad et al., 2024). By integrating AI tools and cultivating AI literacy, college students can benefit from personalized learning experiences that stimulate their interest and creativity.

### The mediating role of problem-solving ability

The results also support Hypothesis 2, confirming that problem-solving ability partially mediates the relationship between AI literacy and creative self-beliefs. This finding aligns with empowerment theory (Zhang S. et al., 2025), information technology can empower individuals through the cognitive pathway such as providing knowledge and skills, coping strategies and improving their ability to managing issues. AI tools reduce cognitive load and automate lower-order cognitive tasks (e.g., data collection and processing), thereby freeing mental resources for higher-order thinking practices and enhancing students’ creativity (Wang et al., 2022). Prior research indicates that AI competency enhances students’ analytical and adaptive problem-solving skills, subsequently improving their general self-efficacy (Lin and Chen, 2024). Empirical studies demonstrate that AI-supported learning environments significantly enhance students’ creative outcomes by fostering idea generation and reducing cognitive barriers (Peng et al., 2025). In such environments, students with high AI literacy can explore different solutions more freely without fear of failure or mistakes, thereby unlocking their creative potential and elevating their creative self-beliefs.

Moreover, high AI literacy enables college students to solve problems more efficiently, fostering greater creative self-beliefs through successful experiences. Cognitive load theory reminds us that learning outcomes are influenced by the allocation of cognitive resources (van Merriënboer and Sweller, 2010). Strong problem-solving ability enables college students to process information more effectively, thereby reducing excess cognitive load and facilitating deeper learning (Koć-Januchta et al., 2022). Students with high AI literacy can use AI tools more efficiently to minimize ineffective cognitive load during the process of problem-solving or dealing with learning tasks (Wang et al., 2025), freeing cognitive resources for creative work and ultimately strengthening creative self-beliefs.

Therefore, AI literacy not only directly enhances creative self-beliefs among college students but also exerts indirect effects via mediating mechanisms. By highlighting the role of problem-solving ability, this study shows how students can transform their AI literacy into better cognitive skills, thereby maintaining their confidence in creative solutions.

### The moderating role of frequency of AI usage

First, supporting Hypothesis 3, this study identified a significant moderating effect of frequency of AI usage on the direct pathway between AI literacy and creative self-beliefs. The results demonstrate that while AI literacy predicts higher CSB across all usage frequencies, its positive effect diminishes with increased usage. Notably, high-frequency AI use substantially attenuates the CSB advantage among high-AI-literacy students. This finding reveals the complex interplay between technological proficiency and behavioral engagement, extending several key theoretical frameworks in educational psychology and human-AI interaction.

According to self-efficacy theory, CSB is significantly associated with autonomy and competence perceptions (Huang and Liaw, 2007). Research indicates that AI adoption enhances employee creativity, primarily through increased creative self-efficacy (Jeong and Jeong, 2024). Moderate use of AI in collaborative work tasks helps reinforce individuals’ positive perception of their own abilities, thereby boosting creative self-beliefs. However, individuals who frequently rely on artificial intelligence to complete creative tasks may experience a decline in autonomy and innovation capacity due to a lack of in-depth engagement with problems. Research indicates that the most significant consequence of over-reliance on GenAI is the decline in university students’ independent thinking ability, reduced innovative thinking capacity, limited self-reflection, and constrained critical thinking skills (Huang and Wu, 2025), significantly undermining their agency in the creative process. Similarly, Yin et al. (2024) identified a double-edged sword effect of AI assistants on employees’ innovative behavior—while they can enhance decision-making efficiency, their high dominance and role erosion may negatively impact employees’ innovative behaviors. For students with low AI literacy, occasional use boosts confidence in creative solutions by providing novelty and some new knowledge. However, frequent use without critical reflection leads to over-reliance, stifles independent thinking, and results in smaller gains in creative self-beliefs.

Conversely, students with high AI literacy gain less creative self-beliefs from frequent AI use. This aligns with the expertise reversal effect: as learners become more skilled, excessive AI support becomes redundant or counterproductive (Kalyuga, 2007). Research further supports this idea, demonstrating that advanced learners may perform worse when given unnecessary guidance (Kalyuga, 2014; Jiang et al., 2023). This explains why highly literate students who use AI sparingly show stronger CSB. Based on the analytical framework of creative self-beliefs (Pavlić et al., 2023), for individuals to effectively employ creativity in their everyday lives, two key elements are essential: namely, creative self-efficacy (CSE), or confidence in their abilities, and a creative personal identity (CPI), the perception of creativity as a valuable personal characteristic (Lebuda et al., 2020). By relying more on independent problem-solving, they engage more deeply, boosting confidence in their creative abilities and valuing creativity as valuable. Limited AI use allows them to leverage it as a supplemental tool—gaining inspiration without dependency. In these cases, creative outcomes are attributed to their own skills, enhancing CSB. In contrast, frequent users outsource cognitive work, reducing opportunities for skill development and creative exploration, which weakens their CPI and ultimately suppresses CSB growth.

Second, our findings support Hypothesis 4, demonstrating that frequency of AI usage moderates the latter path of problem-solving ability’s (PSA) effect on CSB. The study reveals that frequent AI tool usage primarily amplifies the inherent confidence advantage of individuals with high PSA. It functions as a competence amplifier that enhances confidence among those already possessing creative advantages (Ahn, 2024), while showing limited efficacy in boosting CSB for individuals with relatively lower PSA.

Drawing upon attribution theory (Thatcher et al., 2008), significant differences exist in attributional styles between individuals of varying ability levels. Research has demonstrated that individuals with different problem-solving styles exhibit systematic differences in their attribution patterns (Houtz et al., 2007). When succeeding with AI assistance, individuals with high problem-solving agency typically attribute their achievements to internal and controllable factors, such as their effective use of AI tools or their own strategic judgment. These self-directed attributions strengthen their CSB by associating success with personal competence (Xu et al., 2025). On the other hand, those with low PSA (problem-solving ability) more often credit external and uncontrollable factors, such as the AI’s performance, which weakens the confidence-building impact of their successful experiences. Frequent AI use provides both groups with more success and failure experiences. High-PSA individuals’ favorable attributional style enables them to continuously derive efficacy reinforcement from these experiences. Conversely, individuals with low problem-solving ability tend to attribute outcomes disadvantageously, which not only prevents them from gaining benefits from successful experiences but also increases their susceptibility to failure. The more frequently they rely on it, the more their creative self-beliefs decline. These attributional differences may constitute a key psychological mechanism underlying the moderating effect of AI use frequency.

Furthermore, according to self-efficacy theory (Li, 2020), self-efficacy derives from four primary sources: mastery experiences, vicarious experiences, verbal persuasion, and physiological/emotional states. These sources are central to the self-efficacy mechanism in human agency, influencing thought patterns, actions, and emotional arousal (Bandura, 1982). Students’ experiences with AI tools vary based on their problem-solving skills. Those with strong skills often achieve high-quality creative results using AI and see its successes as proof of their own ability, adopting AI’s methods as their own (Hayes et al., 2020; Stolz et al., 2022), therefore enhancing their creative self-efficacy (CSE). In contrast, those with weaker skills tend to credit AI-generated successes to the tool itself rather than their own effort, limiting any boost to their confidence in creative solutions (Chen and Wang, 2023). Some may even compare themselves negatively to AI, increasing self-doubt. Skilled individuals enhance their creative self-beliefs from AI interactions, especially via direct mastery and observational learning (Evans et al., 2020). However, less skilled individuals often fail to gain confidence; instead, their creative personal identity (CPI) may decline due to faulty attributions and negative emotions (Schindler and Bakker, 2020).

In summary, the moderating effect on the direct path shows that frequent AI use diminishes CSB among high-AI-literacy students. Those with high AI literacy but low usage frequency demonstrates superior CSB—their independent problem-solving facilitates deeper cognitive processing that strengthens confidence in creative solutions. However, the latter path’s moderating effect indicates that frequency of AI usage intensifies PSA’s influence on CSB. For high-PSA students, more frequent AI use corresponds with stronger confidence in creative solutions. High-ability individuals use AI as a tool that boosts their abilities. This allows them to focus their saved cognitive resources on more complex creative tasks. These seemingly contradictory findings actually reflect a unified principle: the differential AI effects across populations hinge on competence irreplaceability. Problem-solving ability represents a general competence that AI cannot readily replace, whereas AI literacy constitutes a specific competence where frequent use may threaten high-literacy students’ sense of professional value.

### Theoretical contributions

This study integrates self-efficacy theory, social cognitive theory, attribution theory, and cognitive load theory to construct and validate a moderated mediation model, systematically revealing the intrinsic mechanisms through which AI literacy influences college students’ creative self-beliefs. The findings support the view that AI literacy contributes to creative self-beliefs by improving problem-solving ability, while frequency of AI usage moderates both the direct and latter paths of the mediation model. These discoveries extend current research from multiple theoretical perspectives.

First, grounded in self-efficacy theory (Kleppang et al., 2023), this study confirms the mediating role of problem-solving ability between AI literacy and creative self-beliefs. The results indicate that AI literacy strengthens students’ confidence in their innovative capabilities by providing more successful problem-solving experiences. This finding supports the core tenet of self-efficacy theory that individuals’ beliefs about their abilities stem from mastery experiences. The research reveals how technological literacy in the AI era can reshape self-efficacy by transforming individuals’ competence experiences.

Second, the mediation results deepen our understanding of cognitive development mechanisms in technology-enhanced environments. Through the lens of cognitive load theory, the study elucidates AI literacy’s facilitative effect on problem-solving ability. Students with higher AI literacy can leverage technological tools to reduce lower-order cognitive load, thereby allocating more cognitive resources to higher-order thinking activities. This provides novel evidence about how technology expands human cognitive capacity.

Third, adopting perspectives from both cognitive load theory (van Merriënboer and Sweller, 2010) and the expertise reversal effect (Kalyuga, 2007), this study uncovers the moderating role of frequency of AI usage in the relationship between AI literacy and creative self-self-beliefs. For students with lower AI literacy, their limited technical and conceptual understanding prevents effective utilization of AI to reduce extraneous cognitive load. Conversely, high-AI-literacy students who frequently use AI show diminished creative self-beliefs advantages. This aligns with the expertise reversal effect, suggesting that excessive scaffolding becomes redundant or even disruptive as learners’ expertise develops.

Finally, the moderating effect of AI use frequency on the latter pathway provides empirical support for attribution theory’s proposition about individual differences in attributional styles (Thatcher et al., 2008). High PSA individuals tend to make internal, controllable attributions when succeeding with AI assistance, whereas low PSA individuals more frequently make external, uncontrollable attributions. This illustrates the Matthew effect in ability development during the AI era—the strong get stronger while the weak get weaker (Perc, 2014). Thus, effective human-AI collaboration does not lower the demand for human skills—it actually requires stronger abilities in problem-solving, self-regulation, and critical thinking.

### Implications and limitations

This study yields significant implications for educational practice in higher education. First, AI education should emphasize cultivating students’ AI literacy, particularly their ability to critically evaluate and utilize AI tools, thereby maximizing technology’s potential to enhance creative self-efficacy and creative personal identity in learning activities or solving problems.

Second, our finding that AI literacy positively influences creative self-beliefs through improved problem-solving ability provides empirical justification for AI education reform. Curricular innovations should include interdisciplinary courses like “AI and Innovative Thinking” that integrate technical tool application with domain-specific problem-solving. Pedagogically, project-based learning approaches should be implemented to develop students’ technical integration competencies through problem-solving scenarios, while metacognitive training should be emphasized to foster strategic thinking and prevent over-reliance on AI tools.

Third, the moderating effects of AI usage frequency on both direct and mediated pathways offer crucial insights for establishing usage guidelines: while AI is powerful, its benefits depend on alignment with users’ core competencies. AI education initiatives must concurrently develop fundamental problem-solving skills, metacognitive strategies, and adaptive attribution styles. Differentiated guidance should be provided based on ability levels—restricting usage frequency while emphasizing independent thinking for low-ability students, versus encouraging deep AI integration for high-ability students.

Despite these insights, several limitations should be acknowledged. First, the reliance on self-reported data from college students may introduce both social desirability bias and recall bias. Future studies would benefit from incorporating experimental designs and behavioral measures to validate these findings through methodological triangulation.

Second, this study has notable sampling limitations, including a relatively small sample size and lack of representation across diverse geographical regions, disciplinary backgrounds (e.g., STEM vs. humanities), and educational levels (undergraduate vs. graduate). As our sample was exclusively drawn from Chinese local ordinary undergraduate institutions, cross-cultural comparisons are needed to assess the generalizability of these findings. Consequently, the results should be interpreted with caution and may not extend to broader populations. Future research should systematically examine how disciplinary background, educational level and types of institutions influence AI literacy and creative self-beliefs, while exploring the psychological mechanisms underlying these differences.

Third, the cross-sectional design precludes causal inferences among the studied variables. Future investigations should employ longitudinal designs to establish temporal relationships. Specifically, the multidimensional nature of AI literacy may differentially impact creative self-beliefs, and students from different disciplines may demonstrate distinct patterns of AI tool usage. These considerations highlight important directions for future research, particularly through longitudinal studies that could clarify causal relationships between these variables.

Fourth, the study is limited by its use of a single-item, self-reported measure for frequency of AI usage. This instrument lacks the reliability and validity of a multi-item scale and fails to account for critical qualitative differences in interactions, such as purpose (learning vs. entertainment) and manner (dependent vs. rational use). Future research would benefit from the development of validated questionnaires with higher reliability and validity to enable a more profound exploration of this topic.

Finally, different AI applications (e.g., generative vs. analytical tools) may exert varying effects on students’ creative self-beliefs. The relationship between AI literacy and critical thinking or metacognitive strategies, and their subsequent influence on college students’ creative self-beliefs, represents an important mediating pathway worthy of further investigation.

## Conclusion

Based on an empirical study of 644 university students from central China, this research investigates the impact mechanism of AI literacy on creative self-beliefs. The results demonstrate that AI literacy not only directly enhances students’ creative self-beliefs but also indirectly influences it through improving problem-solving ability. Both the direct path (AI literacy→CSB) and the latter path (PSA→CSB) in the mediation model were significantly moderated by frequency of AI usage. Specifically, students with low AI literacy showed consistently low creative self-beliefs regardless of usage frequency, while high-frequency AI use actually reduced confidence in creative solutions among high-AI-literacy students. Similarly, excessive AI tool use decreased creative self-beliefs for students with lower problem-solving ability, whereas those with higher problem-solving ability maintained stable confidence in creative solutions through effective AI utilization. These findings suggest that AI tool usage follows neither a simple “more-is-better” nor “less-is-better” pattern, but rather produces context-dependent effects based on individuals’ literacy and competence levels. The study provides empirical evidence highlighting the crucial role of coordinated development between technological literacy and cognitive abilities for fostering innovative talents in the AI era.

## Data Availability

The original contributions presented in this study are included in this article/supplementary material, further inquiries can be directed to the corresponding authors.
